# Association Between Conversational Multitasking and Clinician Work Behaviors at a Large US Health Care System: Cohort Study

**DOI:** 10.2196/72768

**Published:** 2025-09-09

**Authors:** Linlin Xia, Daphne Lew, Laura Baratta, Elise Eiden, Sunny Lou, Thomas Kannampallil

**Affiliations:** 1 Washington University in St. Louis, 660 South Euclid Avenue, Campus Box 8054, St Louis, MO, United States, 1 3142737801

**Keywords:** text-based clinician communication, concurrent messaging, electronic health records, attention switching, audit logs, clinician workload

## Abstract

**Background:**

Clinical communication is central to the delivery of effective, timely, and safe patient care. The use of text-based tools for clinician-to-clinician communication—commonly referred to as secure messaging—has increased exponentially over the past decade. The use of secure messaging has a potential impact on clinician work behaviors, workload, and cognitive burden.

**Objective:**

The objective of this study is to investigate the relationship between conversational multitasking—engaging in multiple concurrent, text-based secure messaging conversations—and clinician workload and cognitive burden for inpatient care.

**Methods:**

This observational cohort study included attending physicians, trainee physicians, and advanced practice providers who worked in an inpatient setting at 14 academic and community hospitals affiliated with a large academic medical center in the United States between February and April 2023. The primary exposure was the maximum number of concurrent secure messaging conversations a clinician engaged in during a workday. The co-primary outcomes were total time spent on the electronic health record (EHR; EHR time) and number of switches between patient charts (patient switching) on that workday. Linear mixed-effect models were used to measure the association between the maximum number of concurrent secure messaging conversations, EHR time, and patient switching on the clinician-day level, after adjusting for covariates (age, gender, total secure messaging volume, patient load, and clinical service assignments).

**Results:**

In total, 50,027 clinician-days involving 3232 clinicians (1798 females, 56%; median age 37, IQR 32‐46 y) and 3,556,562 secure messages were included. Median EHR time per day was 307 (IQR 204‐413) minutes, and the median number of patient switches per day was 107 (IQR 60‐176). Compared to clinician-days with no concurrent secure messaging conversations, engaging in a maximum of 2, 3, and 4 or more concurrent secure messaging conversations was associated with an increase in EHR time of 20.3 (95% CI 18.2‐22.4), 38.0 (95% CI 34.9‐41.1), and 54.8 (95% CI 50.6‐58.9) minutes, respectively. Similarly, compared to clinician-days with no concurrent secure messaging conversations, engaging in a maximum of 2, 3, and 4 or more concurrent secure messaging conversations was associated with 14.5 (95% CI 11.3‐17.7), 26.7 (95% CI 21.9‐31.5), and 41.6 (95% CI 35.2‐48.1) additional patient switches, respectively. Stratified analyses showed that trainees experienced the largest increases in EHR time (up to 82.3 min, 95% CI 73.2‐91.4) and patient switches (up to 61.8, 95% CI 54.3‐69.3).

**Conclusions:**

Higher levels of conversational multitasking were associated with increased EHR time and more patient switches in a dose-dependent manner. These results suggest that conversational multitasking may be linked with increased clinician workload and cognitive burden, emphasizing the need for guidelines and interventions to streamline secure messaging use in clinical practice.

## Introduction

With the rapid adoption of mobile phones, personal communication patterns have seen a dramatic shift toward asynchronous, text-based communication [1]. Such changes have also permeated health care settings; asynchronous secure text messaging applications now play a central role in clinical communication [2,3]. Secure text messaging applications allow clinicians to message each other in a Health Information Portability and Accountability Act–compliant environment using mobile and electronic health record (EHR)-integrated platforms [4].

Over the past few years, the use of secure messaging applications has increased nearly 10-fold, underscoring its prominent role in clinician-to-clinician communication [5-7]. The rapid adoption of secure messaging has been driven by a number of factors, including institutional priorities, ease of use, informality in structure, integration with clinical workflow, and asynchronous modality [8-10]. However, despite its widespread adoption, relatively little is known regarding the effect of text-based secure messaging on clinician work activities, workflows, and cognitive burden.

As opposed to synchronous modes of communication (eg, face-to-face, telephone), where participants are engaged in one conversation at a time, asynchronous text-based communication allows individuals to actively engage in multiple concurrent secure messaging conversations at a time. Such an engagement in multiple secure messaging conversations is a form of interleaved multitasking, involving the management of multiple conversations (each message thread can be considered as a separate task) that are progressing in parallel, along with ongoing patient care activities interleaved within them. Prior studies in non-health care settings [11-13] have shown that interleaved multitasking leads to users engaging in the most visible task (often, the interrupting task), with the original task still remaining in memory, causing declines in overall work performance and cognitive burden [14].

Previous research has described the detrimental effects of multitasking and interruptions in health care settings, including increased cognitive workload, delays in task completion, and higher rates of clinical errors [15-19]. Most of these studies relied on observational techniques and small sample sizes. EHR-based measurement using audit logs allows for increasing the scope and scale of such studies [20]. Given the widespread use of secure messaging and its well-described impact on interruptions and fragmentation of work, EHR-based measurement using event and audit logs—by capturing the timing, sequence, and context of user actions—offers an opportunity to study multitasking at scale.

We investigated the relationship between conversational multitasking—concurrently engaging in multiple, ongoing secure messaging conversations—and clinician work behaviors. Specifically, we hypothesized that secure messaging-based conversational multitasking (ie, engaging in multiple concurrent secure messaging conversations) would be associated with increased EHR time and frequency of patient switching among inpatient physicians.

## Methods

### Study Setting

This observational cohort study was conducted at 14 hospitals that were affiliated with BJC HealthCare and Washington University School of Medicine. These hospitals included both academic and community facilities, serving a diverse patient population across urban, suburban, and rural areas in Missouri and Southern Illinois.

All hospitals included in the study used the Epic EHR system. Epic’s Secure Chat, a text-based, EHR-integrated secure messaging platform, has been in use since 2019. Epic’s Secure Chat enables clinicians to send and receive text-based messages with other health care professionals who are part of the same institution directly from within the EHR interface [6]. For both the desktop and mobile versions of the Epic EHR, messages appear in conversational threads.

#### Ethical Considerations

This study was approved by the institutional review board of Washington University School of Medicine with a waiver of informed consent (institutional review board number 202205084). We followed the STROBE reporting guideline for standard reporting in cohort studies [21].

### Participants

This study included all attending physicians, advanced practice providers (APPs; nurse practitioners and physician assistants), and trainee physicians (residents and fellows) who worked in inpatient settings during the 3-month period from February 1, 2023, to April 30, 2023.

### Inclusion and Exclusion Criteria

An inpatient clinician’s workday (ie, a clinician-day) was included if they met the following criteria: (1) the clinician signed at least 1 patient note, (2) placed at least 1 order, and (3) sent or read at least 1 secure message. These criteria were used to ensure that the sample included active clinical workdays (ie, days in which the clinician was involved in direct patient care). This approach, consistent with prior research [22], reasonably excludes days when a clinician was not on service, as signing notes and placing orders are typically associated with direct patient care.

Clinician-days were excluded if they were night shifts, as night shifts have unique workflow dynamics, potentially affecting secure messaging behaviors [23,24]. Although night shift work is common, including such shifts could introduce heterogeneity in workflow patterns and bias the estimates. Night shifts were defined by the clinician’s first EHR login time occurring before 5 AM or after 5 PM (Figure S1 in Multimedia Appendix 1).

Additionally, at the clinician level, we excluded 45 clinicians who lacked demographic information (age and sex) from the analysis. For more information, see the flow diagram, with the inclusion and exclusion criteria, in Figure S2 in Multimedia Appendix 1.

### Data Sources and Data Processing

In total, 2 primary data sources were used for this study: secure messaging metadata and EHR audit logs.

Secure messaging metadata were extracted from Epic’s Clarity database. For each message, we retrieved data including details about the conversation thread, message sender, message recipient, and timestamp that messages were sent and read. Secure messaging metadata and EHR-based audit logs were aggregated at the clinician-day level, defined as a 24-hour period within each calendar day.

EHR audit logs were retrieved from the ACCESS_LOG table in Epic’s Clarity database for the study period. These audit logs, which are required for compliance with Health Information Portability and Accountability Act, provide detailed trails of clinician activities within the EHR. EHR audit logs capture all click-based actions in the EHR that display or modify patient data, offering a comprehensive record of clinical work activities [25,26]. Audit logs have been extensively used in prior research to gain insights into clinician workload, workflows, and clinical behaviors [20,27].

Additionally, clinician-level metadata, including clinician role, clinical service locations (specific clinical settings in which they worked on a particular day), and demographic details were also extracted for analysis.

Finally, clinicians, especially trainees, may have worked in multiple settings over the study period. To account for this, we used the clinical service assignment for each clinician-day based on their EHR login information. When logging into the EHR, clinicians select a “context” for their login, providing them with a customized EHR interface aligned with that clinical service. The most frequent login context for each clinician-day was used as a proxy for their clinical service assignment.

### Measurement of Conversational Multitasking

**Figure 1.**
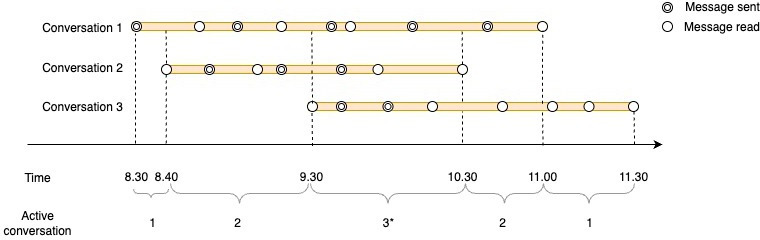
Conceptual characterization for measuring conversational multitasking. Three conversational threads are shown for 1 clinician. For each conversation (horizontal bar), each circle represents a message activity by the user, with double circles indicating a message sent and single circles indicating a message read. The beginning and ending times of each active conversation are determined by the first and last message read or sent in the conversation. The figure depicts 3 secure messaging conversations: Conversation 1 starts at 8:30 AM and ends at 11:00 AM; Conversation 2 starts at 8:40 AM and ends at 10:30 AM; and Conversation 3 starts at 9:30 AM and ends at 11:30 AM. This clinician’s active secure messaging conversations are as follows: between 8:30 AM and 8:40 AM, only conversation 1 is active (0 concurrent secure messaging conversations); between 8:40 AM and 9:30 AM, secure messaging conversations 1 and 2 are active (2 concurrent secure messaging conversations); between 9:30 AM and 10:30 AM, all 3 secure messaging conversations are active (3 concurrent secure messaging conversations); between 10:30 AM and 11:00 AM, secure messaging conversations 1 and 3 are active (2 concurrent secure messaging conversations); and between 11:00 AM and 11:30 AM, only conversation 3 is active (0 concurrent secure messaging conversations). *Indicates the maximum number of concurrent secure messaging conversations for the clinician-day.

To identify conversational multitasking, we first sought to determine the occurrence of concurrent secure messaging conversations during a clinician-day. Towards this end, we defined a conversation as active from the first to the last timestamp of the clinician’s secure message activity within that conversation for each day [28]. For each minute of the clinician-day, we then computed the number of active secure messaging conversations that the clinician was engaged in. For example, Figure 1 illustrates the messaging timeline for a clinician engaged in 3 conversation threads from 9:30 AM to 10:30 AM. Conversation 1 started at 8:30 AM and ended at 11:00 AM; Conversation 2 started at 8:40 AM and ended at 10:30 AM; Conversation 3 started at 9:30 AM and ended at 11:30 AM. The number of concurrent secure messaging conversations at each 1-minute interval was calculated as follows: 8:30 AM to 8:40 AM (0 concurrent); 8:40 AM to 9:30 AM (2 concurrent); 9:30 AM to 10:30 AM (3 concurrent); 10:30 AM to 11:00 AM (2 concurrent); and 11:00 AM to 11:30 AM (0 concurrent). Sensitivity analyses were also performed using 2- and 5-minute intervals to assess the robustness of the findings (Tables S3-S6 in Multimedia Appendix 1).

All data processing and visualization were performed using Python 3.9.10, Pandas 2.2.2, and Matplotlib 3.9.1 [29-31].

### Exposure

The exposure of interest was the level of conversational multitasking, defined as the maximum number of concurrent secure messaging conversations a clinician engaged in during a workday. We categorized the maximum number of concurrent secure messaging conversations for a clinician-day into 4 groups: no concurrent secure messaging conversations, 2 concurrent secure messaging conversations, 3 concurrent secure messaging conversations, and ≥4 concurrent secure messaging conversations.

We conceptualized the maximum number of concurrent conversations as a categorical variable, as the integer values were highly skewed. Moreover, we did not expect the relationship between the number of concurrent conversations and the outcomes of interest to remain constant for each 1-unit increase in concurrency. These specific category boundaries were selected to ensure a relatively balanced distribution of clinician-days across groups, which is important for obtaining a representative picture of the entire population under study and for ensuring unbiased and efficient estimates [32,33]. For instance, as shown in Figure 1, this clinician’s highest number of overlapping secure messaging conversations was 3, so they would be classified in the 3 concurrent secure messaging conversations group. Conversely, if none of the clinician’s secure messaging conversations overlapped during the day, the clinician-day would be categorized in the 0 concurrent secure messaging conversations group.

### Outcome Measures

We used 2 outcome measures: EHR time and patient switching. EHR time, measured in minutes, was calculated from the audit log data by summing the time intervals between successive audit log actions, excluding intervals exceeding 5 minutes, which were considered periods of inactivity. This method for estimating overall EHR time is commonly used as an indicator of clinician workload [34-36]. Prolonged EHR usage has been linked to adverse wellness outcomes, including burnout [34,37,38].

Patient switching was also derived from audit log activities and was computed as the frequency of transitions between patient charts during an uninterrupted EHR session (also referred to as attention switching) [22]. A transition between audit log events in one patient’s chart to another patient’s chart was recorded as a patient switch, unless the interval between the events was greater than 5 minutes. Cognitive science research identifies attention switching as a cognitive burden, which has been associated with an increased risk of errors [36,39].

### Covariates

For each clinician-day, we calculated the total time spent in active secure messaging conversations, representing the cumulative minutes engaged in any messaging. We then determined the percentage of time spent in concurrent secure messaging conversations by dividing the minutes with concurrent secure messaging conversations by the total messaging time.

Other covariates included daily secure messaging volume, patient load, clinician role, clinical service, age, and sex.

### Statistical Analysis

Descriptive statistics were calculated as medians with IQRs or as frequencies with percentages. In total, 2 separate linear mixed effects models were used to investigate the relationship between the maximum number of concurrent secure messaging conversations and both total EHR time and patient switching. Models included the maximum number of concurrent secure messaging conversations as the primary exposure of interest and were adjusted for daily messaging volume, patient load, percentage of time with concurrent secure messaging conversations, clinician role, age, and sex. Models included random intercepts for clinicians to account for repeated observations within clinicians and for clinical service to account for similarities in work patterns within these settings. Because each clinical service was nested within a single institution, this helped to account for institutional-level variation in clinical work patterns. Linear mixed effects models were the preferred statistical approach, as opposed to alternatives such as generalized estimating equations, as it was important to account for clustering among both clinicians and the clinical service where they worked to ascertain the effect estimates after accounting for these variances.

Effect estimates and 95% CIs were calculated to evaluate the expected association between the maximum number of active secure messaging conversations on a clinician-day and the outcome variables. The interaction between the maximum number of concurrent secure messaging conversations on a clinician-day and clinician role was also assessed in a separate model. Based on the significance of the interaction term, 3 separate models, stratified by clinician role, were subsequently run for each outcome variable to explore these associations within specific clinician groups [40]. All statistical analyses were conducted using R (version 4.3.3; R Foundation for Statistical Computing) and RStudio (version 2023.12.1; Posit, PBC) [41,42].

## Results

### Sample Characteristics

This study included 50,027 clinician-days, covering >80 million audit log actions and 3,556,562 secure messages by 3,232 unique clinicians (Figure S2 in Multimedia Appendix 1). The median clinician age was 37 years (IQR 32‐46), and 1798 (56%) were female. Clinician roles were distributed as follows: 54% (1759) were attending physicians, 27% (857) were APPs, and 19% (616) were trainee physicians. The median patient load per day was 7 (IQR 4‐11). Clinicians spent a median of 307 minutes per day on the EHR (IQR 204‐413), with a median of 107 patient switches per day (IQR 60‐176). Detailed descriptive characteristics by clinician role are provided in Table 1.

**Table 1.** Descriptive characteristics of the study sample by clinician role.

	Attending physician (n=1759)	APP^a^ (n=857)	Trainee physician (n=616)
Age (years), median (IQR)	39 (34-50)	39 (34-47)	30 (28-32)
Female, n (%)	741 (42)	740 (86)	317 (51)
Total clinician-days, n	22,277	15,445	12,305
Total EHR^b^ time (minutes/day), median (IQR)	282 (166-406)	319 (232-412)	324 (229-425)
Patient switches (/day), median (IQR)	107 (57-178)	89 (52-137)	141 (85-219)
Patient load (patients/day), median (IQR)	8 (4-14)	6 (4-10)	6 (4-8)
Ordering session (/day), median (IQR)	20 (7-44)	26 (11-48)	23 (9-41)
Secure messaging volume (messages/day), median (IQR)	30 (10-84)	37 (15-79)	51 (20-104)
Total secure messaging time (minutes/day), median (IQR)	155 (25-408)	230 (55-434)	248 (74-456)
Clinician-days with no concurrent secure messaging conversations, n (%)	11,183 (50)	6482 (42)	4211 (34)
Clinician-days with up to 2 concurrent secure messaging conversations, n (%)	4718 (21)	4130 (27)	3386 (28)
Clinician-days with up to 3 concurrent secure messaging conversations, n (%)	2561 (11)	2384 (15)	2248 (18)
Clinician-days with ≥4 concurrent secure messaging conversations, n (%)	3815 (17)	2449 (16)	2460 (20)

a APP: advanced practice provider.

b EHR: electronic health record.

For secure messaging activities, the median volume of secure messages sent and received per day was 37 (IQR 13‐88), with a median of 203 (IQR 42‐430) minutes per day spent on messaging. Concurrent secure messaging conversations occurred on 28,151 (56%) clinician-days, with a median of 34% (IQR 11%‐62%) of messaging time spent in concurrent secure messaging conversations. Further details on secure messaging time across different concurrency levels are shown in Table S7 in Multimedia Appendix 1.

### Concurrent Secure Messaging Conversations and EHR Time

In multivariable analysis, compared to clinician-days with no concurrent secure messaging conversations, clinician-days with a maximum of 2, 3, and 4 or more concurrent secure messaging conversations were associated with an increase in the total EHR time of 20.3 (95% CI 18.2‐22.4), 38.0 (95% CI 34.9‐41.1), and 54.8 (95% CI 50.6‐58.9) minutes, respectively (all *P*<.001).

The interaction between the maximum number of concurrent secure messaging conversations and clinician roles was statistically significant (*P*<.001). Consequently, we conducted a stratified analysis based on the clinician role (attending physician, APP, and trainee physician). Results were maintained within all 3 subgroups, but the associations were strongest among trainee physicians and weakest among APPs (Table 2).

**Table 2.** Effect estimates for the relationship between variables of interest and total electronic health record time, stratified by clinician role.

Dependent variable and variable		Effect estimate (95% CI)		
		Attending physician	APP^a^	Trainee physician
Electronic health record time				
	No concurrent conversation	Reference	Reference	Reference
	Max concurrent secure messaging conversations (=2)	17.37 (14.29 to 20.45)^b^	12.24 (9.16 to 15.32)^b^	30.55 (25.57 to 35.53)^b^
	Max concurrent secure messaging conversations (=3)	29.10 (24.38 to 33.82)^b^	24.79 (20.07 to 29.51)^b^	58.31 (51.36 to 65.27)^b^
	Max concurrent secure messaging conversations (≥4)	40.91 (34.50 to 47.32)^b^	38.32 (32.03 to 44.62)^b^	82.34 (73.21 to 91.46)^b^
	Percent time in concurrent secure messaging conversations (/day)	0.08 (0.01 to 0.15)^b^	–0.05 (–0.12 to 0.02)	–0.15 (–0.25 to –0.04)^b^
	Messaging volume (/day)	0.38 (0.36 to 0.41)^b^	0.37 (0.33 to 0.41)^b^	0.33 (0.29 to 0.37)^b^
	Patient load (/day)	6.96 (6.72 to 7.21)^b^	4.01 (3.74 to 4.27)^b^	9.89 (9.39 to 10.39)^b^
	Clinician age	–0.28 (–0.42 to –0.14)^b^	–0.61 (–1.27 to 0.04)	–0.06 (–1.63 to 1.51)
	Clinician sex (male vs female)	–15.77 (–24.16 to –7.38)^b^	–11.85 (–29.55 to 5.86)	4.12 (–6.67 to 14.92)

a APP: advanced practice provider.

b Indicates *P* value <.05.

Specifically, for trainee physicians, clinician-days with a maximum of 2 concurrent secure messaging conversations were associated with an increase in EHR time of 30.6 minutes (95% CI 25.6‐35.5); clinician-days with a maximum of 3 concurrent secure messaging conversations were associated with an increase of 58.3 minutes (95% CI 51.4‐65.3); and clinician-days with 4 or more concurrent secure messaging conversations were associated with an increase of 82.3 minutes (95% CI 73.2‐91.5), all compared to clinician-days with no concurrent secure messaging conversations.

### Concurrent Secure Messaging Conversations and Patient Switching

In multivariable analysis, compared to having no concurrent secure messaging conversations on a clinician-day, clinician-days with a maximum of 2, 3, and 4 or more concurrent secure messaging conversations were associated with 14.5 (95% CI 11.3‐17.7), 26.7 (95% CI 21.9‐31.5), and 41.6 (95% CI 35.2‐48.1) additional patient switches, respectively.

As with EHR time, there was a statistically significant interaction between the maximum number of active secure messaging conversations and clinician roles for patient switching (*P*<.001). Again, results were maintained in all 3 subgroups, and the strongest association was among trainee physicians (Table 3).

**Table 3.** Effect estimates for the relationship between variables of interest and patient switches, stratified by clinician role.

Dependent variable and variable		Effect estimate (95% CI)		
		Attending physician	APP^a^	Trainee physician
Patient switches				
	No concurrent conversation	Reference	Reference	Reference
	Max concurrent secure messaging conversations (=2)	12.34 (5.47 to 19.20)^b^	8.19 (5.58 to 10.79)^b^	23.25 (19.18 to 27.33)^b^
	Max concurrent secure messaging conversations (=3)	20.53 (9.99 to 31.07)^b^	15.50 (11.51 to 19.50)^b^	43.39 (37.70 to 49.08)^b^
	Max concurrent secure messaging conversations (≥4)	32.35 (18.03 to 46.68)^b^	28.62 (23.30 to 33.94)^b^	61.81 (54.32 to 69.30)^b^
	Percent time in concurrent secure messaging conversations (/day)	0.09 (–0.07 to 0.25)	–0.02 (–0.08 to 0.04)	–0.08 (–0.17 to 0.01)
	Messaging volume (/day)	0.30 (0.23 to 0.36)^b^	0.25 (0.22 to 0.28)^b^	0.32 (0.29 to 0.36)^b^
	Patient load (/day)	3.42 (2.87 to 3.97)^b^	3.32 (3.09 to 3.54)^b^	5.98 (5.57 to 6.39)^b^
	Clinician age	–0.08 (–0.46 to 0.29)	–1.45 (–2.01 to –0.89)^b^	–0.07 (–1.54 to 1.68)
	Clinician sex (male vs female)	–1.54 (–22.32 to 19.23)	6.84 (–8.42 to 22.10)	16.85 (5.79 to 27.91)^b^

a APP: advanced practice provider.

b Indicates *P* value <.05.

For trainee physicians, clinician-days with a maximum of 2 concurrent secure messaging conversations were associated with 23.3 (95% CI 19.2‐27.3) additional patient switches; clinician-days with 3 concurrent secure messaging conversations were associated with 43.4 (95% CI 37.7‐49.1) additional patient switches; and clinician-days with 4 or more concurrent secure messaging conversations were associated with 61.8 (95% CI 54.3‐69.3) additional patient switches, all compared to clinician-days with no concurrent secure messaging conversations.

### Sensitivity Analysis

The concurrency among actively engaged secure messaging conversations was captured at a per-minute level (Tables S1 and S2 in Multimedia Appendix 1). We performed sensitivity analysis with additional time windows (2 and 5 min), and there were no meaningful changes in the effect sizes for both outcomes (Tables S3-S6 in Multimedia Appendix 1).

## Discussion

### Principal Findings

In this large-scale cohort study with >3000 clinicians across more than 50,000 clinician-days, encompassing >3 million secure messages and >80 million audit log actions, we found that increased conversational multitasking was associated with increased EHR time and more patient switches in a dose-dependent manner. This highlights the potential association between conversational multitasking and higher clinical workload and cognitive burden. Notably, higher levels of conversational multitasking were associated with progressively higher clinical workload and cognitive burden.

### Comparisons to Prior Work

A recent, related study, on which the current study was based, found an association between secure messaging volume and both clinician workload and attention switching [22]. The current study examined the impact of inpatient clinicians engaging in concurrent secure messaging conversations and emphasizes the role of conversational multitasking on clinician burden: even after adjusting for messaging volume, higher levels of conversational multitasking were associated with considerably larger increases in both EHR time and patient switches.

This increased burden potentially arises from interleaved multitasking associated with managing active, ongoing messages along with other clinical and EHR-based tasks. Prior research has shown that when users were *prepared* for multiple ongoing tasks (ie, multitasking), their task performance became slower, even if the secondary task was not directly in their view [14]. In other words, the simultaneity of active secure messaging conversations increases the user’s cognitive burden, as the user’s cognitive resources (ie, working memory) are overloaded because of the superimposition of the multiple ongoing messages [43].

The interleaving of work activities has been shown to affect clinician behaviors in a number of settings [15,17,19]. In contrast to most of which included observational techniques with small sample sizes, the current study (with >3000 clinicians) highlights the potential impact of interleaving of clinician work activities on downstream outcomes (ie, clinical work and cognitive burden). Moreover, multitasking using secure messaging may also have patient safety implications. A recent study showed that increased volume of secure messaging may be associated with a corresponding increase in the likelihood of wrong-patient ordering errors [44]. As part of future work, we will investigate the association between conversational multitasking and errors, care delays, or other adverse patient safety events.

Finally, it must be stated that secure messaging has many potential advantages [45]; it is an asynchronous communication mode that allows for the ability to prioritize messages, thus potentially streamlining clinical workflows [46,47]. Prior studies have also found that clinicians perceive secure messaging as being easier to use and better integrated with workflow, potentially improving communication efficiency [4,9,10,47]. However, given the rapid proliferation of the use of digital tools in medicine, it is important to consider their impact on clinicians and their work activities. Increasing use of digital tools has increased clinician work on the EHR, work outside of work, and more concerningly, potentially reduced bedside time with patients and increased clinician burnout [48]. As such, with secure messaging, it is important to carefully consider policies and interventions that can lead towards more efficient use of these digital tools [49,50].

### Limitations

This study has several limitations. First, this study was conducted in the inpatient settings of a single integrated health care system, which may limit its generalizability; however, it did include 14 academic and community hospitals, with a large sample of clinicians (>3000) and their workdays (>50,000). Second, communication needs may also differ based on specific clinical settings and associated workloads (eg, an intensive care unit compared to a hospital ward). We adjusted for this by controlling for the clinical setting and accounting for each clinician’s patient load, but we cannot exclude the possibility of residual confounding. Third, we assumed concurrent conversations contributed to clinician cognitive burden throughout the entire time they were open, and thus we allowed conversations to contribute to concurrency for the entire duration that they were open. However, many conversational threads evolved over extended periods (minutes to hours); as such, it is possible that clinicians may have forgotten about some conversational threads over time, so our conceptualization of concurrency may potentially overestimate the impact of these conversations on cognitive burden. Similarly, the observed association between conversational multitasking, EHR time, and patient switching may be confounded by higher care coordination needs on that workday, as we were unable to directly adjust for patient complexity. However, we could not adjust for patient complexity, primarily because a considerable percentage of the messages (~30%) did not include patient identifiers (eg, a medical record number), making it difficult to link secure messaging conversations to specific patients. Fourth, 45 clinicians were not included because of the lack of their demographic information, potentially introducing selection bias; however, the number of excluded clinicians was small relative to the overall cohort (approximately 1%, 45 out of 3979). Fifth, measurement of the EHR time may be imprecise because it was derived from EHR-based audit logs. However, recent studies have validated that such audit log-based measures of EHR time are closely correlated with observed time spent on the EHR [20,51,52]. Finally, secure messaging was not the only form of communication available; other forms of communication included face-to-face, telephone, and pagers. As such, the overall burden of communication could not be measured. However, as has been shown in recent research, new channels of communication lead to polychronic communication—use of multiple channels for communication (eg, secure messaging and phone)—increasing the overall burden [53].

#### Conclusions

In this large-scale cohort study, we found that increased conversational multitasking with secure messaging was associated with higher clinician workload and cognitive burden, especially among trainees. These findings align with previous work demonstrating that multitasking increases workload, highlighting the potential detrimental effects of secure messaging within a complex health care work environment characterized by frequent interruptions. With limited interventions or strategies to manage the ever-increasing secure messaging volume, future studies should evaluate system-level strategies to mitigate clinician burden.

## Supplementary material

10.2196/72768 Multimedia Appendix 1 Supplementary materials and appendix.

## Abbreviations

APP: advanced practice provider
EHR: electronic health record
