# Supplementary material for: Association Between Conversational Multitasking and Clinician Work Behaviors at a Large US Health Care System: Cohort Study
Source: J Med Internet Res. 2025 Sep 9;27:e72768. doi: 10.2196/72768 (PMC12419866; doi:10.2196/72768)

**Supplementary Material**

^1^Linlin Xia, BS^*^, ^2^Daphne Lew, PhD^*^, ^3^Laura R. Baratta, BS, ^4^Elise Eiden, MS, ^2,4^Sunny S. Lou, MD, PhD^**^, ^2,4,5^Thomas Kannampallil, PhD^**, §^

**Supplemental Table 1.** Effect estimates for the relationship between variables of interest and EHR time (1-minute intervals).

| **Dependent variable** | **Variable** | **Effect Estimate (95% CI)** | **P value** |
| --- | --- | --- | --- |
| EHR time | No concurrent secure messaging conversation | *Reference* | |
|  | Max concurrent secure messaging conversations (=2) | 20.32 (18.23, 22.41) | < 0.001 |
|  | Max concurrent secure messaging conversations (=3) | 38.00 (34.89, 41.11) | < 0.001 |
|  | Max concurrent secure messaging conversations (≥4) | 54.77 (50.61, 58.94) | < 0.001 |
|  | Secure messaging volume | 0.37 (0.35, 0.39) | < 0.001 |
|  | Patient load | 6.52 (6.34, 6.70) | < 0.001 |
|  | % time concurrent secure messaging conversations | -0.05 (-0.10, -0.00) | 0.035 |
|  | Provider role: Physician vs. APP | -53.42 (-61.44, -45.39) | < 0.001 |
|  | Provider role: Trainee vs. APP | -15.01 (-24.65, -5.36) | < 0.001 |
|  | Clinician age | -0.33 (-0.47, -0.18) | < 0.001 |
|  | Clinician sex: Male vs. Female | -9.52 (-16.25, -2.79) | 0.006 |

**Supplemental Table 2.** Effect estimates for the relationship between variables of interest and patient switches (1-minute intervals).

| **Dependent variable** | **Variable** | **Effect Estimate (95% CI)** | **P value** |
| --- | --- | --- | --- |
| Patient switches | No concurrent secure messaging conversation | *Reference* | |
|  | Max concurrent secure messaging conversations (=2) | 14.51 (11.30, 17.72) | < 0.001 |
|  | Max concurrent secure messaging conversations (=3) | 26.72 (21.93, 31.51) | < 0.001 |
|  | Max concurrent secure messaging conversations (≥4) | 41.64 (35.22, 48.05) | < 0.001 |
|  | Secure messaging volume | 0.30 (0.27, 0.33) | < 0.001 |
|  | Patient load | 3.86 (3.59, 4.14) | < 0.001 |
|  | % time in concurrent secure messaging conversations | -0.01 (-0.09, 0.06) | 0.724 |
|  | Provider role: Physician vs. APP | 13.94 (-0.89, 28.78) | 0.020 |
|  | Provider role: Trainee vs. APP | 25.61 (7.56, 43.66) | 0.020 |
|  | Clinician age | -0.20 (-0.48, 0.08) | 0.157 |
|  | Clinician sex: Male vs. Female | 6.55 (-6.04, 19.14) | 0.308 |

**Supplemental Table 3.** Effect estimates for the relationship between variables of interest and EHR time (2-minute intervals).

| **Dependent variable** | **Variable** | **Effect Estimate (95% CI)** | **P value** |
| --- | --- | --- | --- |
| EHR time | No concurrent secure messaging conversation | *Reference* | |
|  | Max concurrent secure messaging conversations (=2) | 20.71 (18.57, 22.84) | < 0.001 |
|  | Max concurrent secure messaging conversations (=3) | 37.86 (34.68, 41.04) | < 0.001 |
|  | Max concurrent secure messaging conversations (≥4) | 55.47 (51.24, 59.69) | < 0.001 |
|  | Secure messaging volume | 0.37 (0.35, 0.39) | < 0.001 |
|  | Patient load | 6.53 (6.35, 6.71) | < 0.001 |
|  | % time in concurrent secure messaging conversations | -0.06 (-0.11, -0.02) | 0.008 |
|  | Provider role: Physician vs. APP | -52.30 (-60.36, -44.24) | < 0.001 |
|  | Provider role: Trainee vs. APP | -13.69 (-23.36, -4.02) | < 0.001 |
|  | Clinician age | -0.32 (-0.47, -0.18) | < 0.001 |
|  | Clinician sex: Male vs. Female | -9.49 (-16.24, -2.73) | 0.006 |

**Supplemental Table 4.** Effect estimates for the relationship between variables of interest and patient switches (2-minute intervals).

| **Dependent variable** | **Variable** | **Effect Estimate (95% CI)** | **P value** |
| --- | --- | --- | --- |
| Patient switches | No concurrent secure messaging conversation | *Reference* | |
|  | Max concurrent secure messaging conversations (=2) | 14.67 (11.37, 17.96) | < 0.001 |
|  | Max concurrent secure messaging conversations (=3) | 26.48 (21.56, 31.40) | < 0.001 |
|  | Max concurrent secure messaging conversations (≥4) | 41.98 (35.43, 48.53) | < 0.001 |
|  | Secure messaging volume | 0.30 (0.27, 0.33) | < 0.001 |
|  | Patient load | 3.89 (3.60, 4.17) | < 0.001 |
|  | % time in concurrent secure messaging conversations | -0.02 (-0.09, 0.05) | 0.589 |
|  | Provider role: Physician vs. APP | 14.06 (-0.91, 29.03) | 0.017 |
|  | Provider role: Trainee vs. APP | 26.29 (8.10, 44.49) | 0.017 |
|  | Clinician age | -0.19 (-0.47, 0.09) | 0.176 |
|  | Clinician sex: Male vs. Female | 6.87 (-5.84, 19.57) | 0.290 |

**Supplemental Table 5.** Effect estimates for the relationship between variables of interest and EHR time (5-minute intervals).

| **Dependent variable** | **Variable** | **Effect Estimate (95% CI)** | **P value** |
| --- | --- | --- | --- |
| EHR time | No concurrent secure messaging conversation | *Reference* | |
|  | Max concurrent secure messaging conversations (=2) | 22.01 (19.80, 24.22) | < 0.001 |
|  | Max concurrent secure messaging conversations (=3) | 39.41 (36.12, 42.70) | < 0.001 |
|  | Max concurrent secure messaging conversations (≥4) | 57.51 (53.17, 61.86) | < 0.001 |
|  | Secure messaging volume | 0.37 (0.35, 0.39) | < 0.001 |
|  | Patient load | 6.54 (6.35, 6.72) | < 0.001 |
|  | % time in concurrent secure messaging conversations | -0.10 (-0.15, -0.05) | < 0.001 |
|  | Provider role: Physician vs. APP | -50.28 (-58.46, -42.11) | < 0.001 |
|  | Provider role: Trainee vs. APP | -12.70 (-22.48, -2.91) | < 0.001 |
|  | Clinician age | -0.32 (-0.46, -0.17) | < 0.001 |
|  | Clinician sex: Male vs. Female | -10.55 (-17.39, -3.70) | 0.003 |

**Supplemental Table 6.** Effect estimates for the relationship between variables of interest and patient switches (5-minute intervals).

| **Dependent variable** | **Variable** | **Effect Estimate (95% CI)** | **P value** |
| --- | --- | --- | --- |
| Patient switches | No concurrent secure messaging conversation | *Reference* | |
|  | Max active conversation (=2) | 14.70 (11.24, 18.16) | < 0.001 |
|  | Max active conversation (=3) | 26.26 (21.09, 31.42) | < 0.001 |
|  | Max active conversation (≥4) | 41.97 (35.15, 48.78) | < 0.001 |
|  | Secure messaging volume | 0.30 (0.27, 0.33) | < 0.001 |
|  | Patient load | 3.89 (3.60, 4.18) | < 0.001 |
|  | % time in concurrent secure messaging conversations | -0.03 (-0.11, 0.04) | 0.414 |
|  | Provider role: Physician vs. APP | 15.65 (0.37, 30.93) | 0.017 |
|  | Provider role: Trainee vs. APP | 26.51 (8.01, 45.01) | 0.017 |
|  | Clinician age | -0.19 (-0.47, 0.09) | 0.193 |
|  | Clinician sex: Male vs. Female | 6.23 (-6.72, 19.18) | 0.346 |

**Supplemental Table 7.** Secure messaging time characteristics by concurrency level.

|  | Clinician-days with no concurrent secure messaging conversation | Clinician-days with up to 2 concurrent secure messaging conversations | Clinician-days with up to 3 concurrent secure messaging conversations | Clinician-days with ≥4 concurrent secure messaging conversations |
| --- | --- | --- | --- | --- |
| Count | 21,876 | 12,234 | 7,193 | 8,724 |
| Percentage of messaging time at maximum concurrency level | 100% | 8% | 3% | 8% |
| Messaging time with no concurrent secure messaging conversation  (messages/day) | 34  (7, 112) | 197  (108, 311) | 223  (141, 317) | 157  (90, 236) |
| Messaging time with 2 concurrent secure messaging conversations (messages/day) | N/A | 18  (5, 58) | 113  (58, 190) | 232  (153, 323) |
| Messaging time with 3 concurrent secure messaging conversations (messages/day) | N/A | N/A | 13  (4, 41) | 97  (50, 156) |
| Messaging time with ≥4 concurrent secure messaging conversations (messages/day) | N/A | N/A | N/A | 46  (11, 144) |

Abbreviation: N/A, not applicable.

**Supplemental Figure 1.** Distribution of first login times for clinician-days.

This figure illustrates the frequency of clinicians' first login times across the study period, highlighting the natural inflection points used to distinguish between day and night shifts.


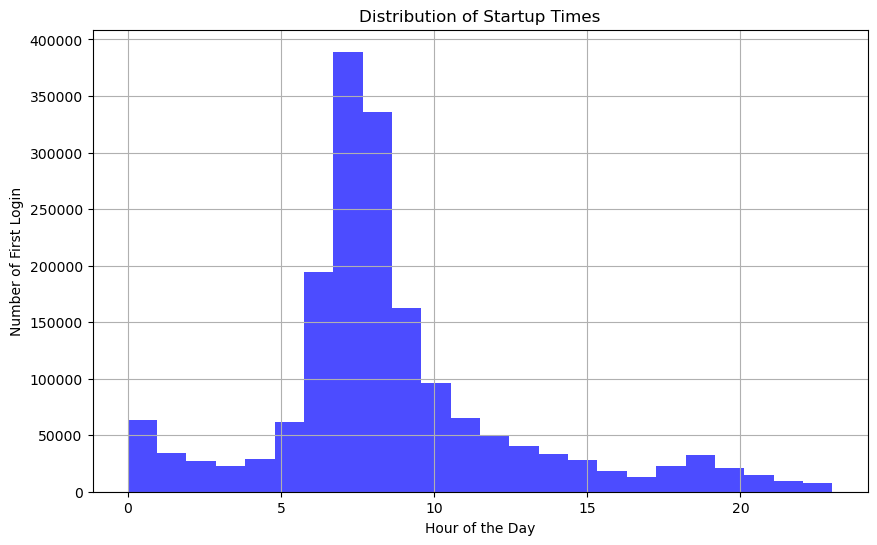


**Supplemental Figure 2.** Flow diagram for inclusion and exclusion of clinician-days.


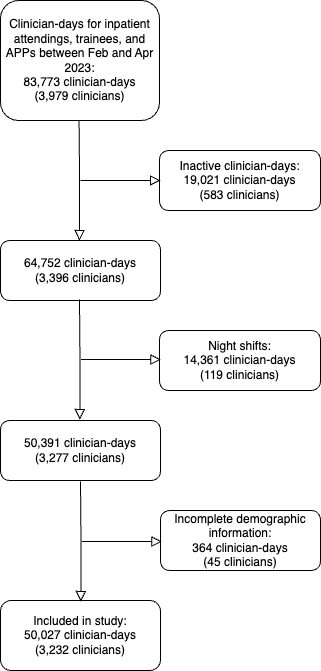

Supplement: Multimedia Appendix 1 [file jmir-v27-e72768-s001.docx]
